# Short-Term Dispersal Response of an Endangered Australian Lizard Varies with Time of Year

**DOI:** 10.1371/journal.pone.0106002

**Published:** 2014-08-22

**Authors:** Mehregan Ebrahimi, C. Michael Bull

**Affiliations:** 1 School of Biological Sciences, Flinders University, Adelaide, South Australia, Australia; 2 Department of Biology, Shiraz University, Shiraz, Fars Province, Iran; Macquarie University, Australia

## Abstract

Dispersal is an important component in the demography of animal populations. Many animals show seasonal changes in their tendency to disperse, reflecting changes in resource availability, mating opportunities, or in population age structure at the time when new offspring enter the population. Understanding when and why dispersal occurs can be important for the management of endangered species. The pygmy bluetongue lizard is an endangered Australian species that occupies and defends single burrow refuges for extended periods of time, rarely moving far from the burrow entrance. However, previous pitfall trapping data have suggested movement of adult males in spring and of juveniles in autumn of each year. In the current study we compared behaviours of adult lizards each month, over the spring-summer activity period over two consecutive field seasons, to provide deeper understanding of the seasonal dispersal pattern. We released adult pygmy bluetongue lizards into a central area, provided with artificial burrows, within large enclosures, and monitored the behaviour and movements of the released lizards over a four day period. There was a consistent decline in time spent basking, amount of movement around burrow entrances, and rates of dispersal from the central release area from early spring to late summer. Results could be relevant to understanding and managing natural populations and for any translocation attempts of this endangered lizard species.

## Introduction

Animal dispersal is triggered by a range of seasonally variable ecological factors [1], [2]. Animals may disperse to reduce kin competition (for example during natal dispersal) [3], to reduce the risk of predation [4], [5], or to escape from a seasonally harsh environment [6]. They can also disperse to increase the chance of finding an appropriate mate [7]–[9]. The advantages for an individual disperser are balanced by energetic costs, costs of time spent during dispersal, costs of increased risk from exposure, and costs of lost opportunities that might have been available if the individual had not dispersed [10]. All of these costs might also vary with the time of the year.

In conservation management of endangered species, dispersal can be an important process. Dispersal between small populations allows gene flow and reduces the risks of inbreeding within populations [11], [12]. However, for populations that persist in small isolated fragments of previously contiguous habitat, dispersing individuals may risk never locating another suitable habitat patch, or at least may risk prolonged exposure before locating suitable habitat. An imbalance between the number of individuals dispersing from a population that occupies a habitat fragment, and the number migrating into the population could lead to local population decline. Assisted migrations or translocations between isolated populations are a management strategy sometimes suggested to enhance gene flow without the risks of natural dispersal [13]. But the dispersal tendencies of the managed species may then diminish the effectiveness of this strategy if animals released into a population site disperse away from the release area, without contributing to the local population [14]. Thus an understanding of the dispersal behaviour, and how that varies with season, will be integral to management strategies for endangered species.

We investigated dispersal related behaviour at different times of year in an endangered Australian skink, the pygmy bluetongue lizard (*Tiliqua adelaidensis*). Populations of this lizard are now restricted to a few remnant patches of native grassland in a small geographic region in the mid-north of South Australia. This region has a Mediterranean climate, with cool wet winters and hot dry summers, and pygmy bluetongue lizard activity is restricted to the austral spring and summer (September–March) [15]. The species is normally solitary and individual lizards spend most of their time associated with their single entrance, vertical burrows, which have been constructed by mygalomorph and lycosid spiders [15], [16]. They use the burrows as refuges from predators and from climatic extremes, and they partially emerge to sit at the burrow entrance to bask and to ambush passing invertebrate prey [15], [16]. Although they aggressively defend their burrows from conspecifics, that defence is restricted to a region of less than 15 cm from the burrow entrance [17]. In natural populations they rarely emerge fully from their burrows, and their movements away from the burrow entrance are normally limited to defecation and catching prey. Occasionally adults move to find new burrows, males move to locate female partners in spring, and neonates and juveniles disperse to establish their own burrows in late summer [18], [19]. When lizards move from their burrows they become vulnerable to predation from birds and snakes [20], and they risk moving out of the small patches of suitable habitat.

In this study we compared short-term dispersal related behaviours of lizards when they were released into novel burrows at different times within their activity season. We had three aims. The first was to document how dispersal behaviour of adult lizards varied across the lizard activity season under a standard set of conditions. We considered these results would help to interpret data from field populations and to understand the overall dynamics of burrow occupancy. Our second aim was to provide advice to conservation managers of this lizard to allow them to interpret population density surveys, and alert them about times in the season of highest risk from exposure to predators. Our third aim was to gain insights into the potential success of management programs of assisted migration or translocation, where an initial target would be to have released animals remain close to the area where they were released. This target might be more easily achieved during a time of the year when they are more sedentary.

## Methods

The data that we use in this paper have already been reported as the control treatments of a series of experimental studies over the austral spring and summer of 2009–2010 and 2010–2011. Those studies investigated how variable conditions such as food availability, vegetation density and burrow density influenced the behaviour of the lizards and their tendency to disperse soon after being moved into a novel burrow. The methods have been previously reported in papers describing this series of studies [21]–[24]. In summary, we captured four male and four female pygmy bluetongue lizards from each of two populations near Burra, South Australia (33° 42′ S, 138° 56′ E) in September 2009, and used these same 16 lizards in all trials over the next 16 months. We were prevented from using different lizards in different trials by the capture permit conditions for this endangered species. The time over which we ran the trials included two successive spring and summer periods, when these lizards are normally active in the field. Lizards were held in individual plastic boxes (52.5 cm×38 cm×31 cm) before and between trials in a room with ambient conditions, and they were fed crickets and meal worms every third day. After the last trials in the 2009–2010 spring and summer field season, lizards were kept in the Animal Care Unit of Flinders University, Adelaide in a 25°C room with an initial 12∶12 LD light regime. We reduced temperature and light gradually to 15°C and 10∶14 LD over the austral winter, and then brought light and temperature back to 25°C and 12∶12 LD by the following spring for the second field season of our trials.

For the trials, we established four circular cages (15 m diameter) in a line, about 5 m apart in the grounds of Monarto Zoo, South Australia (35° 06′ S, 139° 09′ E). Cages had 1 m high-galvanised iron walls and bird-proof wire roofs. We divided each cage into three areas; a central 4 m diameter circular area with artificial burrows provided where lizards were released, a 5 m wide matrix around the central area, considered to be unsuitable habitat as it did not have any refuge burrows or vegetation, and a ring, 0.5 m wide, around the inside cage perimeter with burrows that trapped any lizards that dispersed from the central area. We constructed artificial burrows from 30 cm lengths of 3 cm diameter wooden dowling with a 2 cm diameter hole drilled out of the centre. Artificial burrows were hammered into the ground until they were flush with the ground surface. Pygmy bluetongue lizards readily accepted these artificial burrows both in field populations [25] and in our cages [26]. We distributed 41 artificial burrows in the central area of each cage as previously described [21], one in the middle, and then 8, 16 and 16 burrows in three concentric rings. In this arrangement, each burrow was spaced an average 63 cm (SE = 0.01) from the next nearest burrow. We also distributed 30 artificial burrows evenly around the perimeter ring of each cage as refuges for lizards that dispersed from the central area. We ran a series of experimental trials during two spring and summer periods, from October 2009 to March 2010 and from October 2010 to January 2011. Each trial lasted four days, and each tested the impact of some experimental manipulation within the central cage area, for instance manipulation of food level [21], of vegetation density [22], or of the arrangement of the burrows [24]. In most trials two cages were used to apply an experimental treatment, and two were used as controls. The comparisons between experimental and control treatments have been documented in previous publications. In this paper we consider only the control treatment cages which were identical in all trials, with burrow conformation as described above, with no additional food supplementation, and with all vegetation in the central area cut to ground level. Normally there were two cages with these control treatments in each trial but sometimes only one. Thus the only differences among the different trials were the month (Oct–March) and field season (2009/10 or 2010/11) when the trials were run, and the increasing experience of the same group of lizards with the trial conditions over successive trials. In total we ran 17 trials across the two field seasons, with 28 control treatment cages considered in this analysis (Table 1).

**Table 1:** The number of trials and the number of cages with the control treatment in each trial in each month of each field season.

	Field seasons			
	2009/2010		2010/2011	
Month	No. trials	Cages/trial	No. trials	Cages/trial
October	1	2	1	1
November	2	2	1	2
December	1	2	1	2
January	2	1	2	2
February	3	1	0	0
March	3	2	0	0

In each trial we released four lizards in each cage (two males and two females) and confined them to the central area for one day using a 20 cm high black plastic wall [23]. We then removed the wall and observed lizard behaviour and movements for the next four consecutive days. To observe behaviour, we suspended four surveillance cameras (Longse: LICS23Hf, 3.5 mm lens) above each cage with a combined field of view that covered the central 4 m diameter area. Cameras recorded lizard behaviour during each day of trials from 0700 to 1800 h onto a 16 channel h.264 DVR (ESW26), powered by four 12 V batteries.

From our filmed records we derived seven behavioural parameters in each trial that allowed us to compare lizard behaviour among different months. In natural populations, pygmy bluetongue lizards spend most of their time associated with a single refuge burrow, retreating down the burrow to escape predators and climatic extremes, or sitting at the burrow entrance to bask and to ambush passing invertebrate prey [16]. Although lizards, in the field, occasionally emerge fully to bask, to capture prey, or to defecate, they rarely move further than a few centimetres from their burrow entrance [15], [23], [27]. The behaviours described below relate to this burrow centred focus of activity.

1) Activity time (h d^−1^) was defined as the period from the first time the lizard head emerged from its burrow to the last time that lizard retreated completely into its burrow on that day. 2) Basking time (min h^−1^) was defined as the amount of time when the lizard had at least partially emerged and remained at the entrance of its burrow. We called this basking because the lizard was exposed to solar radiation, but an additional function of this behaviour may have been to ambush passing invertebrate prey. We divided the total minutes spent basking each day by 11 (total hours of filming) to calculate the basking time as minutes per hour. 3) Number of movements around burrow. In some cases lizards fully emerged from their burrow, moved about, usually for a very short distance, and then retreated to the same burrow. These movements included lizards that just walked around the burrow entrance, lizards that basked while fully emerged, and lizards that moved away from the burrow entrance for defecation or foraging for prey. We recorded the number of these movements by each lizard on each day. 4) Number of burrow changes. In some other cases lizards fully emerged from their burrow and moved around to choose another burrow in the central area. We recorded the number of burrow changes for each lizard on each day. 5) Distance moved. If a lizard had moved to a different burrow within the central area during a day we measured the distance moved as the direct line distance between the burrow the lizard was in at the start of the day to the burrow it was in at the end of the day. 6) Number of dispersals. We recorded the mean number of cases per day when a lizard left the central area, moved across the habitat matrix, and was subsequently discovered occupying a burrow in the perimeter region. There could be no more than four dispersal events (from the four lizards) on any one day, but sometimes lizards subsequently dispersed themselves back to the central area and could be recorded dispersing again on another day. This behavioural parameter was not recorded in trials in February and March of the first season. 7) Number of fights. When two lizards approached each other on the ground surface, they always showed some agonistic interaction, either with the lizards scuffling together, or with one running away from the other. We defined all of these interactions as fights, and recorded the number of fights per lizard on each day.

In the trials, when a lizard moved into the perimeter area, it left the field of view of the cameras, and we had incomplete information about its behaviour on that day. For analyses, we derived one value of each behavioural parameter from the four lizards in each cage in each trial, using the average over all four days, from all lizards with complete data on each day.

Although the same 16 lizards were used in all 17 sets of experimental trials, in each trial we selected different combinations of four lizards for each of the control cages, and we treated each of the 28 sets of control cage results as independent replicates.

First we explored whether ambient thermal conditions during the trials influenced any of the behavioural parameters. There is an overall tendency for air temperatures to increase from spring to summer in South Australia. We derived average, minimum and maximum temperatures over each four day trial from temperature records at Pallamana Aerodrome weather station (35° 04′ S, 139° 13′ E), 10 km from Monarto Zoo. We then used one way ANOVAs to determine if the mean temperatures experienced differed among months, and Pearson correlations to examine the relationships between those temperatures and the average behavioural parameter values during each trial.

Next we took the results from October to January in each season, and conducted a two-way analysis of variance (ANOVA) for each behavioural parameter, with factors month (Oct–Jan) and field season (2009/10 or 2010/11). A significant month×season interaction would indicate that any behavioural change across months differed between the two seasons. One interpretation of that might be that they were becoming more familiar with the trial arenas in the second season, and adjusting their behaviours with experience. This might suggest that behavioural changes could be explained by increasing experience with the conditions rather than by any effect of season. Our results (see below) did not show any significant month×season interaction effects, so for the final analysis we pooled all trials across the two field seasons to derive mean values per month and used nested one-way ANOVAs to investigate the effect of month of release for each behavioural parameter. We used the month of the trial as a fixed factor with replicate control cages nested within months. We used the Bonferroni test for post hoc comparisons between pairs of months.

### Ethics Statement

The study was conducted according to the guidelines of the Flinders University Animal Welfare Committee (approval no E206) in compliance with the Australian code of practice for the use of animals for scientific research and was conducted under Permit G25011 from the South Australian Department of Environment, Water and Natural Resources. This permit allowed us to captured 16 specimens (8 male and 8 female) from two populations near Burra (33° 42′ S, 138° 56′ E) and transfer them to Monarto Zoo (35° 06′ S, 139° 09′ E) and use them for the experiments described above. All 16 lizards were captured by hand after luring them from their burrows with mealworms, according to the method described by Fenner et al. [28]. This capture method was approved according to the guidelines in our permits. Monarto Zoo, the site of the experimental investigations, is privately owned by Zoos SA.

## Results

**Figure 1:**
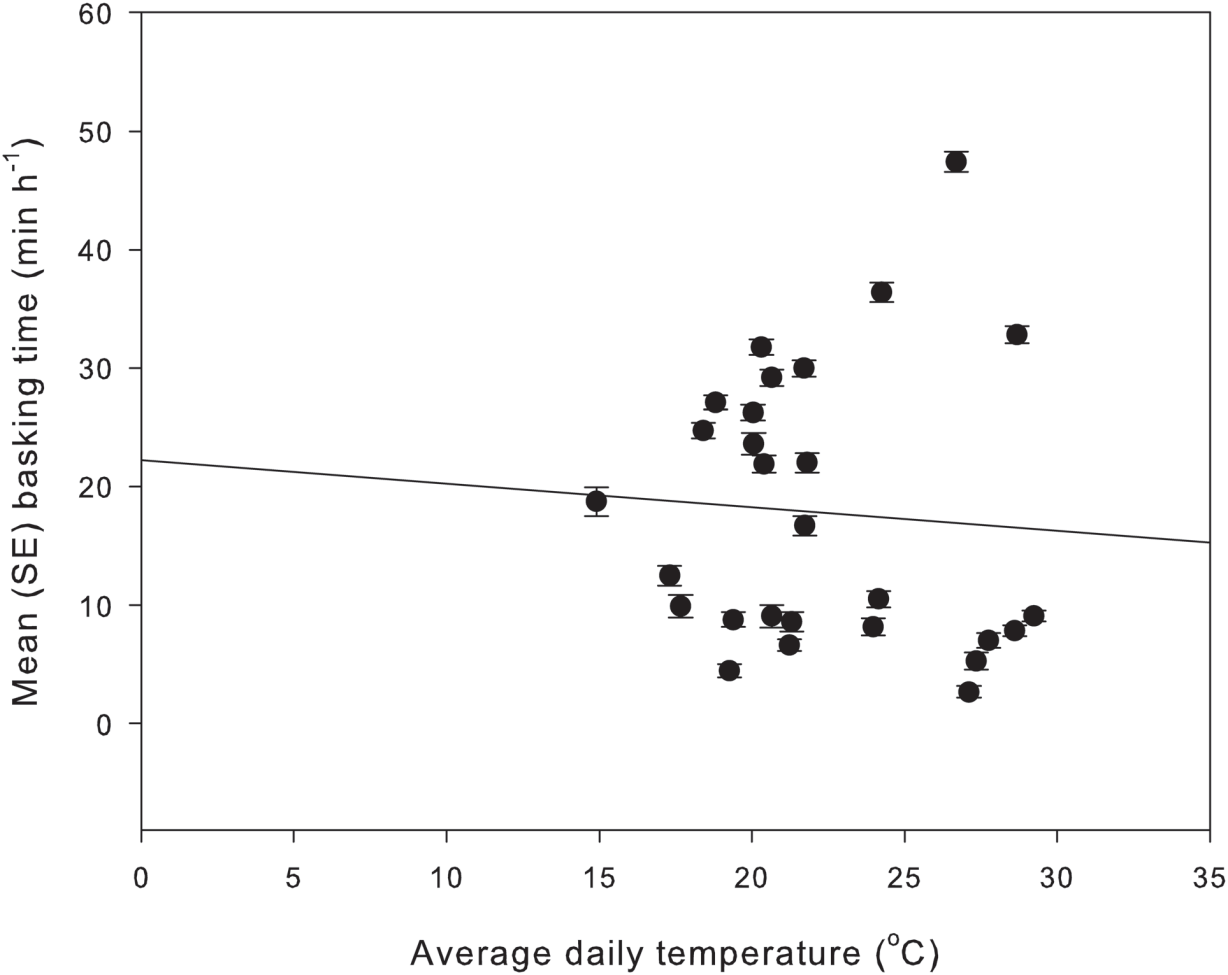
Relationship between the mean basking time of all lizards in a cage over a four day trial and the mean of the average daily temperature over the four day trial. The solid line indicates the significant negative correlation.

Although there was a wide range of ambient temperatures experienced over the 17 trials (Fig. 1), there were no significant difference among months for the ambient thermal conditions we measured during the trials, either for the average (F_5,9_ = 0.32, p = 0.87), minimum (F_5,9_ = 3.39, p = 0.13), or maximum temperatures (F_5,9_ = 0.44, p = 0.80). Only one behavioural parameter was significantly correlated with temperature. Lizard basking time decreased when temperature increased (average temperature: r = −0.434, p = 0.021 (Fig. 1); minimum temperature: r = −0.710, p<0.001; maximum temperature: r = −0.518, p<0.005). There were no significant interactions between month and field season for any of the behavioural parameters (Table 2). The significant effect of month for six of those behaviours over the period October to January remained consistent between the two field seasons 2009/10 and 2010/11.

**Table 2:** Analyses of variance (ANOVA) considering the effect of month (Oct–Jan) and field season (2009/2010 and 2010/2011) on each of seven behavioural parameters, using months when trials were run in both field seasons.

Behavioural parameter	Field season			Month			Field season×month		
	df	*F*	p	df	*F*	p	df	*F*	p
Activity time	1, 12	1.68	0.21	3, 12	4.73	0.021*	2, 12	0.70	0.51
Basking time	1, 12	0.23	0.64	3, 12	4.33	0.027*	2, 12	3.16	0.08
Number of movements around burrows	1, 12	1.38	0.26	3, 12	16.99	0.001*	2, 12	0.36	0.70
Number of burrow changes	1, 12	1.02	0.33	3, 12	8.09	0.003*	2, 12	0.77	0.48
Distance moved	1, 12	0.03	0.85	3, 12	2.03	0.163	2, 12	0.91	0.42
Number of dispersals	1, 12	0.01	0.91	3, 12	7.49	0.005*	2, 12	0.44	0.52
Number of fights	1, 12	0.63	0.44	3, 12	4.28	0.028*	2, 12	0.45	0.64

Values with *indicate significant effects (p<0.05).

**Figure 2:**
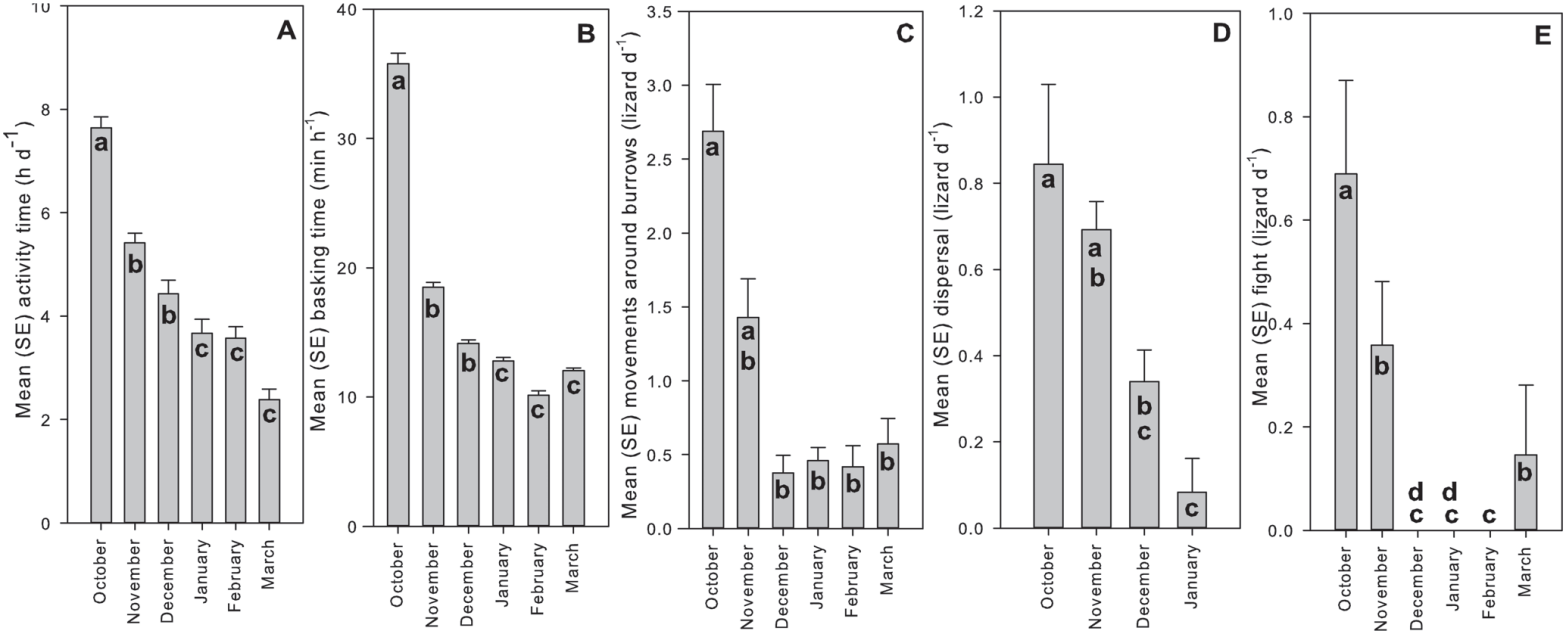
Mean and one standard error for five behavioural variables from trials in each month. Bars with different lower case letters were found to be significantly different in posthoc bonferroni pairwise comparisons. A) Mean activity time, B) Mean basking time, C) Mean number of movements around the burrow, D) Mean number of dispersals (no data available for February and March), E) Mean number of fights.

When the data were pooled across field seasons, and data from February and March 2010 were included in the analyses, five behavioural parameters retained significant differences among months (Table 3). For all five of those parameters there was a consistent trend for decreasing values as the field season progressed from spring to summer (Fig. 2). Lizards spent a shorter period of the day active (Fig. 2a), they basked for less time (Fig. 2b), moved around their burrows less often (Fig. 2c), dispersed from the central area less often (Fig. 2d), and were involved in fewer fights (Fig. 2e) as the season progressed from spring (October) to late summer (March).

**Table 3:** Analyses of variance (ANOVA) considering the effect of month on each behavioural parameter over all trials.

Behavioural parameter	Month			Nested factor (Cage(Month))		
	df	*F*	p value	df	*F*	p value
Activity time	5, 16	47.23	0.001*	6, 16	0.43	0.673
Basking time	5, 16	33.02	0.001*	6, 16	1.26	0.333
Number of movements around burrows	5, 16	5.03	0.036*	6, 16	2.55	0.062
Number of burrow changes	5, 16	1.42	0.336	6, 16	5.51	0.003*
Distance moved	5, 16	0.63	0.682	6, 16	2.67	0.054
Number of dispersals	3, 9	17.81	0.007*	6, 16	0.79	0.561
Number of fights	5, 16	591.3	0.001*	6, 16	0.08	0.966

Values with *indicate significance (p<0.05).

## Discussion

The data from our series of trials showed a consistent pattern across the two field seasons of monthly differences in the behaviours of pygmy bluetongue lizards. The lack of any significant month×season interaction effects suggests that those differences represent a real monthly change in behaviour, rather than an accumulating familiarity by our captive lizards for the experimental conditions. In that case we would have expected different levels of activity or behaviour in the spring of the second field season than in the first season. We cannot completely discount the possibility that the lizards had reset their experience level during the winter break, and that increasing familiarity with the experimental conditions accounted for the behavioural changes each season. However, we considered that a less likely explanation of the trends we observed, given their consistency with field observations.

The field observations showed similar trends of higher levels of activity and movement in spring than later in summer. All of the incidental observations of predation of exposed lizards have occurred in October or November [20], [29] and Schofield et al. [18] found that the number of adult pygmy bluetongue lizards captured in pitfall traps was highest in the spring, coinciding with the time when mating behaviour had previously been observed [30]. They suggested that lizards are most likely to leave their burrows and move around (to be trapped by pitfalls) at the time when they are seeking mating partners [18]. This may be reflected by the higher rates of movements and burrow changes in spring in our trials. Changes in some of our other behavioural parameters may be also correlated with these movements. For instance there may have been fewer fights in summer than spring because the lizards spent less time active, and moved less around their burrow entrances, so there were fewer opportunities for two individuals to encounter each other.

We considered whether the behavioural changes may have been temperature related. Temperatures in southern Australia generally increase from spring to summer, and one behavioural parameter, basking time, showed significant negative correlations with the mean ambient temperature, and with the minimum and maximum temperatures measured over the four day trials. This was consistent with the reduction in basking time with month within each field season. Perhaps lizards spent shorter periods emerged when it was warmer, to avoid overheating. However, other behaviours that also significantly declined over successive months, were not significantly related to temperature during the trials. Furthermore, there were no significant differences in temperature among months in the four day periods when individual trials were run, for any of the three temperature parameters that we considered. We concluded that, while some behaviours may be affected by the ambient temperature conditions, consistent behavioural changes in the lizards occurred from month to month independent of ambient temperature variation.

### Management implications

Whatever the causal explanation of the trends we observed, the results have two implications for a conservation management program for this lizard species. First, within existing populations, behavioural insights derived from our results can alert managers about when lizards are at greatest risk from predators and from dispersal away from managed population sites. Dispersal has greater significance on populations of a species like the pygmy bluetongue lizard that persist in small fragments of previously more continuous native grassland habitat. Field populations of pygmy bluetongue lizards decline in size over the spring and summer period [31], and part of that attrition is likely to result from dispersal, that, in a fragmented landscape, is not balanced by migration in from other populations [32]. If artificial burrows are added to provide additional refuges for dispersing lizards, and to reduce both the time exposed on the surface, and the chance of moving away from suitable habitat, the seasonal population decline might be averted [33]. Our behavioural studies now indicate when this management intervention will be most effective.

The second implication of our results involves planning for possible translocations. The period immediately after release, while individuals become adjusted to and familiar with the novel release environment, is probably critical for the success of any translocation [34], [35]. Animals may be stressed from the combined procedures of handling, holding and transportation [36], and they will be unfamiliar with refuge and foraging resources at the release site [37]–[39], but if they remain close to where they are released until they have settled into the new conditions, then there is a chance they will stay [23], [39], [40].

Three aspects of our results suggest that lizards translocated later in the summer may have more chance of successfully establishing at the translocation site. First, lizards were less frequently active, moved about around their burrow entrance less, and spent less time basking at the burrow entrance later in the season. All of these behavioural changes would reduce the exposure of lizards to potential predators at the new site. Second, lizards interacted agonistically less often later in the season, avoiding increased levels of stress from intraspecific interactions. And third, the lizards dispersed less often from the central release area in our trials later in the season.

Ultimately the success of a translocation depends on the longer term responses of the translocated animals, although the first major first step is to prevent short-term dispersal away from the release area. This study has suggested that, for pygmy bluetongue lizards, the time of release could play a significant role in determining the possibility of dispersal from the translocation area, and that later in the activity season is a better time.

There are two broader conclusions from this study. One is that behavioural changes across different seasons might be critical for managing existing and translocated populations of endangered species. For our study system the remarkable seasonal differences in adult activity behaviour, from the current study and from results that have already been reported [18], have suggested that the time of mating might be important. Other reptiles and amphibians with narrow mating seasons, might have similar windows of time when they are more active, more exposed to predators, more likely to disperse away from secure habitat, and are subject to more stress. Conservation intervention, to reduce risks and stress related factors, may be most appropriate at these times. Counter to this, the monitoring of population density and individual body condition in these amphibians and reptiles, and the capture of individuals from source populations for translocation, might be easiest at the times when they are most active. Among mammals and birds, that have more stable social structures across longer periods of time, and where dispersal is often among subadults and juveniles, an equivalent period may be less obvious. The second conclusion is that understanding the basic behaviour of the target species is essential for optimizing management and translocation success [41], [42].
